# Organically Modified Silica Nanoparticles Are Biocompatible and Can Be Targeted to Neurons In Vivo

**DOI:** 10.1371/journal.pone.0029424

**Published:** 2012-01-03

**Authors:** Farda Barandeh, Phuong-Lan Nguyen, Rajiv Kumar, Gary J. Iacobucci, Michelle L. Kuznicki, Andrew Kosterman, Earl J. Bergey, Paras N. Prasad, Shermali Gunawardena

**Affiliations:** 1 Department of Biological Sciences, The State University of New York at Buffalo, Buffalo, New York, United States of America; 2 Institute of Lasers, Photonics and Biophotonics, The State University of New York at Buffalo, Buffalo, New York, United States of America; Columbia University, United States of America

## Abstract

The application of nanotechnology in biological research is beginning to have a major impact leading to the development of new types of tools for human health. One focus of nanobiotechnology is the development of nanoparticle-based formulations for use in drug or gene delivery systems. However most of the nano probes currently in use have varying levels of toxicity in cells or whole organisms and therefore are not suitable for *in vivo* application or long-term use. Here we test the potential of a novel silica based nanoparticle (organically modified silica, ORMOSIL) in living neurons within a whole organism. We show that feeding ORMOSIL nanoparticles to Drosophila has no effect on viability. ORMOSIL nanoparticles penetrate into living brains, neuronal cell bodies and axonal projections. In the neuronal cell body, nanoparticles are present in the cytoplasm, but not in the nucleus. Strikingly, incorporation of ORMOSIL nanoparticles into the brain did not induce aberrant neuronal death or interfered with normal neuronal processes. Our results in Drosophila indicate that these novel silica based nanoparticles are biocompatible and not toxic to whole organisms, and has potential for the development of long-term applications.

## Introduction

The use of nanotechnology in biomedical research is expected to have a major impact on human welfare, due to the development of new types of diagnostic and therapeutic tools. However, the same properties that make nanoparticles so attractive for development in nanomedicine could also prove deleterious when nanoparticles interact with cells within a living organism. Indeed, there are a number of nano probes (quantum dots (QDs) [1], upconverting nanophosphors [2], and luminophore-containing nanoparticulate carriers such as liposomes [3], polymersomes [4] ceramic [5], [6], or polymeric [7] nanoparticles), which claim to be efficient theranostic tools for *in vivo* imaging and also have potential for use in therapeutic applications. However, many of these nano probes have long-term toxicity issues that dilute their efficiency for long-term use *in vivo*.

Recently, interest has generated in the use of new silica-based nanoparticles for imaging. Silica-based nanomaterials such as sol-gel, colloidal, mesoporous and organically modified silica (ORMOSIL) nanoparticles, have shown promising potential in biotechnological applications such as nanoprobes for actively targeted optical imaging [8], [9]. These inert, optically transparent materials can be conjugated with any desired fluorophore (visible or NIR), leading to the generation of robust, fluorescent nanoparticles [10], [11]. Furthermore, these nanoparticles are porous and have tunable pore size, so they can be incorporated with bioactive molecules such as enzymes, genetic materials, and chemotherapeutic drugs. They can also be tagged with a peptide for a specific biological function [12], [13], thus allowing targeting to specific cell types or tissues. In addition, the chemistry of silica provides the opportunity for a variety of surface functionalities (hydroxyl/amino/thiol/carboxyl groups, [14], [15]), which can be used to attach biotargeting molecules. Previously ORMOSIL nanoparticles conjugated with fluorophores and targeting ligands have been used in optical imaging of tumor cells *in vitro* [16], [17]. These particles have also been tested for potential use in photodynamic therapy, and as targeted optical probes for imaging of pancreatic cancer cells in *in vitro* experiments [18], [19], [20]. However, to date the biocompatibility, bio distribution and toxicity of ORMOSIL has not been tested in living organisms.

Drosophila melanogaster is an ideal model system to rapidly test novel nanoparticles that have potential therapeutic applicability for human diseases. The Drosophila genome is highly conserved when compared to higher organisms. Many homologous genes encoding proteins that cause human diseases have been identified [21]. Many human neuronal disease models such as Alzheimer's disease, Huntington's disease and Parkinson's disease have also been developed in the fly [22]. Furthermore, cellular and developmental mechanisms are extensively conserved between flies and humans. Thus studies on the uptake of new therapeutic agents and their lethality in Drosophila can be well correlated to mammalian systems. The developmental and behavioral aspects of Drosophila are also documented, thus making the fly an informative and adaptable model to investigate a wide variety of toxicological endpoints relevant to human biology. Recently, a number of powerful assay methods have been developed in Drosophila to test developmental and behavioral toxicology of potential therapeutic agents. Results from these tests have been used to prioritize further testing in rodents, and some of these agents have later been translated into human clinical trails [23], [24], [25].

Here we report the *in vivo* characterization of a novel silica based nanoparticle, ORMOSIL in Drosophila. We provide evidence that these nanoparticles are biocompatible and can be effectively used in living organisms for a long period of time. We show that these nanoparticles are not toxic to the developing organism and do not disrupt any major cellular functions. ORMOSIL nanoparticles readily incorporate into living neuronal cells. Incorporation of ORMOSIL does not activate aberrant neuronal death pathways, nor does it affect the long distance transport pathway (axonal transport), which is essential for the growth, maintenance and survival of all neurons. Taken together, our results suggest that ORMOSIL nanoparticles can be safely used in living neuronal tissues and in living organisms with minimal effects on normal cellular functions. This is the first study that thoroughly investigates the biocompatibility of this class of nanoparticles throughout an organism's development and in neuronal cells, which is an important consideration for these particles to be eventually translated into use in humans.

## Results

### Physiochemical properties of ORMOSIL nanoparticles

**Figure 1:**
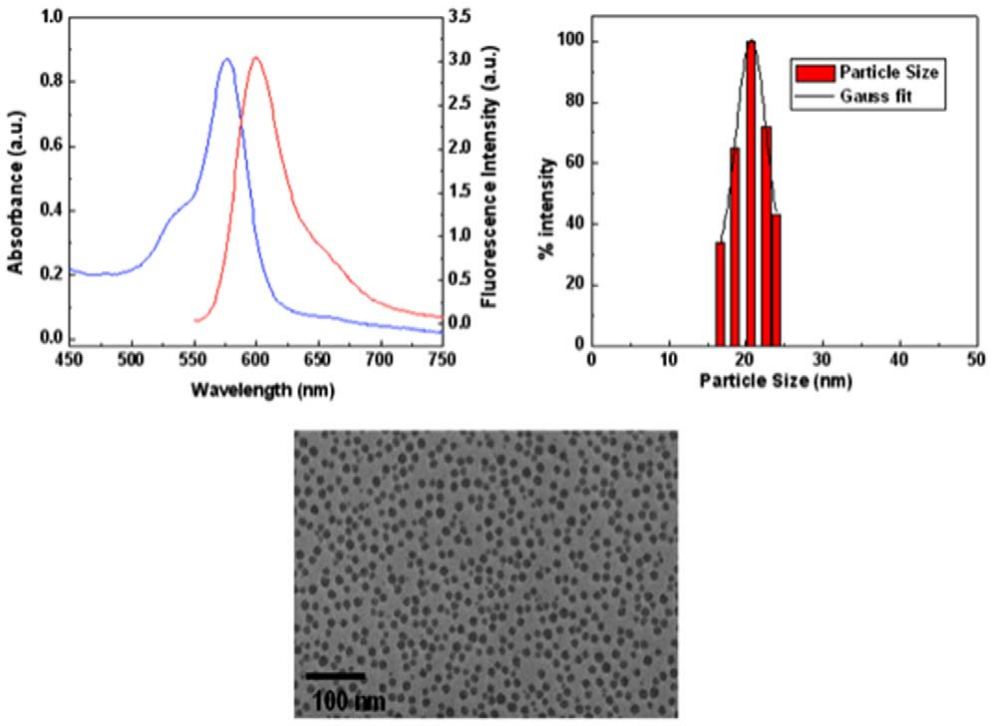
Physiochemical properties of ORMOSIL nanoparticles. (**A**) The absorption and emission spectra for rhodamine-ORMOSIL (^R^ORM) particles. The typical emission band of rhodamine is λ_max_ 600 nm. (**B**) The ORMOSIL nanoparticle size distribution profile as measured by the dynamic light scattering (DLS) method indicates that the diameter of ORMOSIL nanoparticles are on average 20 nm. (C) A transmission electron microscope (TEM) image of ORMOSIL nanoparticles. Bar = 100 nm.

To test the biocompatibility, bio distribution, and the eventual fate of ORMOSIL nanoparticles *in vivo* following systemic delivery, we used fluorescently conjugated ORMOSIL. We use both unconjugated and conjugated (receptor and peptide-conjugated) rhodamine-ORMOSIL nanoparticles (^R^ORM). The synthesis of the ^R^ORM nanoparticles was adapted from our previous work with minor modifications [8], [ 26] and the nanoparticles were characterized by various methods as described in figure 1. Figure 1, shows the basic properties of ^R^ORM including its absorption and emission spectra. ^R^ORM emits at a typical emission band for rhodamine, which is λ_max_ 600 nm (Figure 1A). The size distribution profile as measured by dynamic light scattering (DLS) method (Figure 1B) and transmission electron microscopy (TEM, Figure 1C) indicate that the diameter of ^R^ORM particles are on average 20 nm, much smaller than the average diameter of synaptic vesicles, which are on average about 40 nm [27]. For receptor-conjugated ^R^ORM, we use the transferrin receptor (TfR, TfR-^R^ORM). Transferrin bound TfR receptor is transported into the cell by endocytosis in a vesicle [28] and is the major route of cellular iron uptake. This cellular uptake pathway is currently being exploited for site-specific delivery of anticancer drugs and therapeutic genes into proliferating malignant cells [29]. For peptide-conjugated ^R^ORM (peptide-^R^ORM), we used 15aa of the C-terminus of the amyloid precursor protein (APP). APP transports a subclass of vesicles in the anterograde direction down the axon [30]. Both conjugation strategies involved the use of simple Ethyl-3-(3-dimethylaminopropyl) carbodiimide (EDCI) chemistry using the functional amine groups present on the surface of ^R^ORM and carboxyl group of APP. Recent work has shown that 15aa of the C-terminus of APP is sufficient for APP to bind to the anterograde molecular motor for axonal transport [31]. Both TfR-^R^ORM and peptide-^R^ORM particles have similar diameters as unconjugated ^R^ORM, and these particles resemble the size of biological vesicles. As a control for fluorescence and to see the applicability of ORMOSIL nanoparticles with different fluorophores, we tested cy5 conjugated ORMOSIL (^cy5^ORM, Figure S1, Table 1) and observe similar effects as ^R^ORM (see below). In addition, although in many of our experiments (Table 1) we have used ^cy5^ORM we have focused on ^R^ORM as the main formulation.

**Table 1:** Summary of ORMOSIL feeding experiments in Drosophila.

	Water	Buffer	^R^ORM 0.2 mg/ml	^R^ORM 0.4 mg/ml	^R^ORM 1 mg/ml	Peptide-^R^ORM 0.2 mg/ml	Peptide-^R^ORM 0.4 mg/ml	Peptide-^R^ORM1 mg/ml	TfR-^R^ORM 0.2 mg/ml	TfR-^R^ORM 0.4 mg/ml	TfR-^R^ORM 1 mg/ml	^cy5^ORM 1 mg/ml
**Larvae**												
Locomotion defects	NO	NO	NO	NO	NO	NO	NO	NO	NO	NO	NO	NO
Viable	YES	YES	YES	YES	YES	YES	YES	YES	YES	YES	YES	YES
**Pupae**												
Viable	YES	YES	YES	YES	YES	YES	YES	YES	YES	YES	YES	YES
**Adult flies**												
Flying defects	NO	NO	NO	NO	NO	NO	NO	NO	NO	NO	NO	NO
Paralysis	NO	NO	NO	NO	NO	NO	NO	NO	NO	NO	NO	NO
Viable	YES	YES	YES	YES	YES	YES	YES	YES	YES	YES	YES	YES

### Drosophila development is not affected by ORMOSIL particles

**Figure 2:**
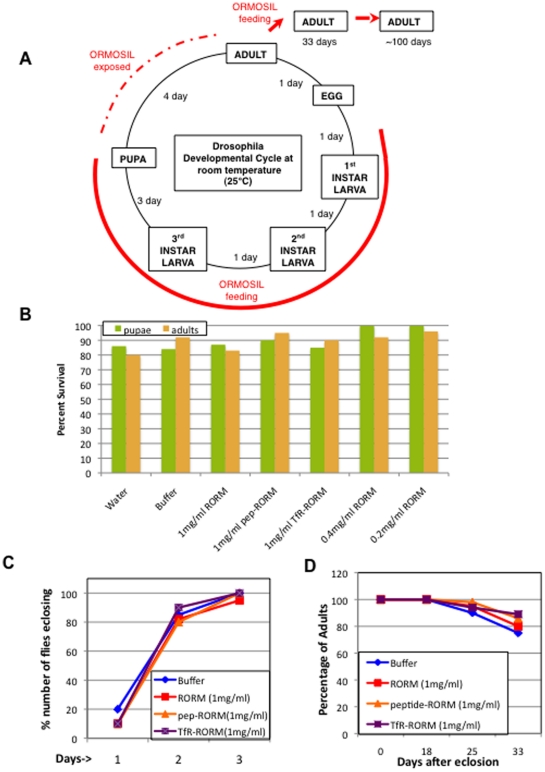
Drosophila development or survival is not affected by ORMOSIL nanoparticles. (A) The Drosophila melanogaster developmental cycle and ORMOSIL feeding scheme. At room temperature (25°C) larvae hatch from embryos and undergo three larval stages (1^st^, 2^nd^ and 3^rd^ instar) each lasting approximately 1 day. After the 3^rd^ instar larval stage larvae begin to pupate. Pupation lasts for 4 days. Once adult flies eclose they can live for over 100 days. Embryos were collected and placed in food vials containing different forms of ORMOSIL, buffer or water. Larvae hatch from embryos and immediately begin feeding. All feedings were done until 3^rd^ instar larvae pupated. The red line depicts the extent of oral administration of ORMOSIL during Drosophila development. The dashed red line depicts the duration of ORMOSIL exposure. Adult flies that eclosed from each feeding condition were collected and placed in new food vials containing the same food that they fed on as larvae. The adult flies continued to feed on different forms of ORMOSIL, buffer or water from the day they eclosed (red arrows). We quantified the survival of adult flies for 33 days (D), while we monitored the survival of adult flies for over 100 days. Note that Drosophila was continuously fed ORMOSIL for a total of 39 to 139 days while they were exposed to ORMOSIL for a total of 43 to 143 days. (B) Oral administration of ORMOSIL nanoparticles has no affect on the survival of Drosophila. The survival of larvae fed on different forms of ORMOSIL was quantified to evaluate survival of pupae and adults. Feedings on buffer (1XPBT) and water were done as controls. All larvae were allowed to develop into pupae and adults, and the total numbers of pupae and adults were counted, normalized and graphed as percentage of survival (Y-axis). No significant difference is seen in the survival of pupae (green) and adults (orange) between the different forms of ORMOSIL, buffer or water fed conditions. (C) Oral administration of ORMOSIL has no affect on the developmental time of when adult flies eclose from pupae. No significant difference is seen in the total number of adult flies that eclosed from ^R^ORM, Peptide-^R^ORM and TfR-^R^ORM (1 mg/ml) or buffer (1XPBT) fed larvae on 3 different days. On each day the total number of flies were counted, normalized and graphed as percentage of number of flies eclosing vs day 1, 2 or 3. (D) Oral administration of ORMOSIL nanoparticles has no affect on the survival of adult flies. The number of adult flies in each food vial was counted on day 0, 18, 25 and 33. The total number of flies in each vial was normalized to 100 and the survival of adult flies was graphed as a survival curve. Y axis represents the percentage of adults that survived. On day 0 flies were collected, counted and transfered to new food vials. On day 18 flies in each of the vials were again counted. No significant difference in survival was seen in adult flies fed on ^R^ORM, Peptide-^R^ORM, TfR-^R^ORM (1 mg/ml) or buffer. Similarly, on day 25 and 33 flies were again counted and no significant difference in the rate of survival of adults fed on ^R^ORM, Peptide-^R^ORM, TfR-^R^ORM or buffer was seen. Note that these flies fed on ORMOSIL or buffer for a total of 39 days (6 days as larvae+33 days as adults), while they were exposed to ORMOSIL or buffer for a total of 43 days (6 days as larvae+33 days as adults+4 days exposed as pupape).

To test the biocompatibility of ORMOSIL in a living organism, we used Drosophila as a model system. Because of its rapid life cycle and relatively low maintenance costs, Drosophila provides us with an ideal system to test initial toxicological effects and obtain dosage information for potential new therapeutic agents. We evaluated the effects of ORMOSIL throughout the life cycle of the fly (Figure 2A). Unlike vertebrate model systems, Drosophila is very amenable to rapid analysis since its life cycle is short [32], [33] and thus effects on an organisms' entire development can be quickly analyzed. Briefly, at room temperature (25°C), 1^st^ instar larvae hatch within 24 hours after fertilization. Hatched larvae undergo three larval stages, each stage lasting about a day. After the 3^rd^ larval stage, metamorphosis and pupation occur, and after 4–5 days of pupation adult flies eclose. Adult flies typically live for over 100 days, which also allows studies on aging to be easily performed.

To test the toxicity of ORMOSIL in a whole organism we fed ^R^ORM (and ^cy5^ORM, data not shown) to Drosophila larvae that had just hatched and we continued to feed ORMOSIL through the developmental cycle of the fly (Figure 2A, red, a total of 39 feeding days and a total of 43 days of exposure). To date no other animal study has fed nanoparticles or followed their survival for such a long duration of time and through an organisms' entire life cycle.

For our feeding experiments, equal numbers of embryos were placed in food-containing vials. We used three different forms of ORMOSIL particles, unconjugated ^R^ORM and two forms of conjugated-^R^ORM (TfR-^R^ORM and peptide-^R^ORM). We used Instant Medium (Carolina Biological), which is a commonly used food for Drosophila in the form of dry flakes to mix all ORMOSIL formulations (at concentrations of 0.2, 0.4 and 1 mg/ml) for all feedings. As controls, food was also mixed with the buffer used to dilute ORMOSIL, (1× phosphate buffered saline with 0.1% triton (1XPBT)) and water. In each food vial containing the different ORMOSIL formulations (three forms of ^R^ORM each at concentrations of 0.2, 0.4 and 1 mg/ml), buffer, or water, larvae were allowed to hatch and were allowed to grow until the larvae pupated (6 days). During the three larval stages, larvae actively gorged and only fed on food containing nanoparticles, buffer or water. During the larval stages, larvae were assayed to determine if feeding on nanoparticles caused locomotion defects. We failed to observe any crawling or locomotion defects for larvae fed on the three forms of ORMOSIL, and these larvae were comparable to control conditions (buffer and water fed larvae, data not shown, Table 1). Larval viability changes were also assessed and no significant difference in the survival of larvae fed on ORMOSIL was seen compared to buffer or water fed conditions (data not shown, Table 1). Thus our observations indicate that feeding on ORMOSIL had no affect on the behavior or on the viability of larvae (Table 1). Further, unconjugated and conjugated ^R^ORM fed larvae were undistinguishable from each other.

To test the affect of ORMOSIL on pupal development, we counted the number of pupae that pupated from ORMOSIL, buffer and water fed larvae (see methods). Larvae were allowed to pupate in the same food vials that they fed on as larvae and the pupae were counted. No effect was seen in the number of larvae that pupated between ORMOSIL, buffer or water fed conditions (Figure 2B, Table 1). Figure 2B depicts the normalized percentage of pupae for all feeding conditions from three different feeding experiments. Further, no abnormality was seen in pupation of ORMOSIL fed larvae compared to buffer or water fed larvae. Note that since larvae were allowed to pupate in the same food vials as they fed on, pupae were still exposed to the same food conditions (4 days of exposure as pupae).

To determine ORMOSIL effects on survival to adulthood, flies were collected as they eclosed and were placed in new food vials containing food laced with the corresponding nanoparticle (form and dose), buffer or water and examined (see methods). We failed to see any abnormality in flies that survived to adults between ORMOSIL, buffer or water fed conditions (Figure 2B). No significant difference in the percentage of flies that eclosed from pupae for the different feeding conditions were observed (Figure 2B, Table 1). In addition, there was no abnormality in the developmental time of when adults emerged from pupae, with almost all adults emerging on the 3^rd^ day for all feeding conditions (Figure 2C). Thus unconjugated or conjugated ^R^ORM appear to be benign and show good biocompatibility during the entire life cycle of the fly (10 days).

We continued to test the biocompatibility of ORMOSIL during adulthood by evaluating adult flies. To test this, the flies that eclosed from larvae fed on ORMOSIL or control conditions (buffer and water) were counted and placed in new food vials containing the corresponding food source (day 0) and the survival of adult flies were monitored for a total of 33 days. Note that during these 33 days adult flies were feeding on the same food source that they fed on as larvae. On day 18, 25, and 33, we counted the number of flies that had died in each vial and plotted the percentage of survival on a survival plot (Figure 2D, see methods). During these 33 days no difference was seen in the survival of adult flies feeding on ORMOSIL or buffer (Figure 2D, blue, Table 1). Furthermore, we also failed to see any behavioral defects. Adult flies were evaluated for flying defects and for the bang sensitive phenotype, a characteristic phenoytpe that depicts problems in synapse transmission. Typically flies that show a bang sensitive phenotype become paralyzed after the flies are given a mechanical shock such as a bang of the vial on a bench/table [34], [35]. We failed to observe any of these problems in flies fed on ORMOSIL and their behavior was undistinguishable from buffer or water fed flies (data not shown, Table 1). We continued to follow these flies as they aged for over three months (approx 100 days) and found no difference in their survival or in their behavior, regardless of the feeding regime (data not shown). In our studies Drosophila was continuously fed ORMOSIL for a total of 39 to 139 days while they were exposed to these particles for a total of 43 to 143 days. Thus continuous feeding of ORMOSIL (unconjugated or conjugated) in Drosophila did not affect the behavior or the survival of adult flies.

### The short-term survival of dissected larvae is not affected by ORMOSIL nanoparticles

**Figure 3:**
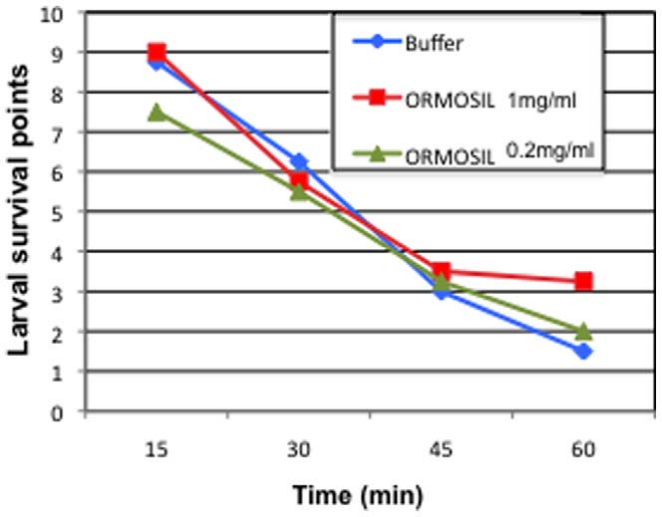
The short-term survival of dissected living larvae is not affected by ORMOSIL nanoparticles. Dissected living larvae were treated with ^R^ORM (1 mg/ml and 0.2 mg/ml are shown) or buffer and larval survival was quantified by evaluating the twitching phenotype. We assigned “survival” values or survival points according to the extent of twitching observed when the larva was stimulated with a forceps. As described in the text, the more the larva twitched, the higher the “survival” value, indicating that the dissected larva was alive [43], [44], [45]. The extent of twitching was recorded every 15 minutes for a total of 60 minutes. No significant difference was seen between the survival of ORMOSIL treated or buffer treated dissected living larvae.

To further evaluate the toxicity of ORMOSIL nanoparticles, we applied ORMOSIL to dissected living larvae and evaluated their survival. We routinely use this larval dissection procedure in our laboratory to study the larval nervous system [36] and dissected larvae can survive under physiological conditions (in dissection buffer) for at least 180 minutes. Under physiological conditions larvae were treated with ORMOSIL for 10 minutes immediately after dissection. The same three doses of ^R^ORM (0.2, 0.4 and 1 mg/ml) that we used for our feeding experiments were used for larval treatment. ^R^ORM-treated larvae were then washed with dissection buffer and immediately evaluated. We found that application of ^R^ORM directly to dissected larvae did not cause any adverse effects to the dissected living larvae. Similarly application of ^cy5^ORM or conjugated ^R^ORM (peptide-^R^ORM or TfR- ^R^ORM) also did not show any adverse effects (data not shown). Thus the survival of all ORMOSIL treated larvae (unconjugated or conjugated) was comparable to buffer treated larvae (Figure 3).

We quantified the survival of dissected living larvae by evaluating the twitching phenotype. We assigned a “survival” value according to the extent of twitching observed when the larva was stimulated with a forceps. The twitching phenotype or the twitch tension is a commonly used phenotype in Drosophila larvae and evaluates nerve-stimulated contractions of larval body-wall muscles [37], [38], [39]. The more the larva twitched or contracted, the higher the “survival” value, indicating that the dissected larva is alive. Using this quantification method we found no obvious difference in the survival of larvae between the three different doses of ORMOSILor buffer treated larvae (Figure 3). The steady decline in twitching that we observed for all conditions can be attributed to a decline in the proper physiological conditions because the dissection buffer was not replaced every 20–30 minutes [40]. Taken together our observations indicate that application of ORMOSIL does not affect the short-term survival of dissected living larvae.

### ORMOSIL nanoparticles incorporate into living larval and adult neuronal tissues

**Figure 4:**
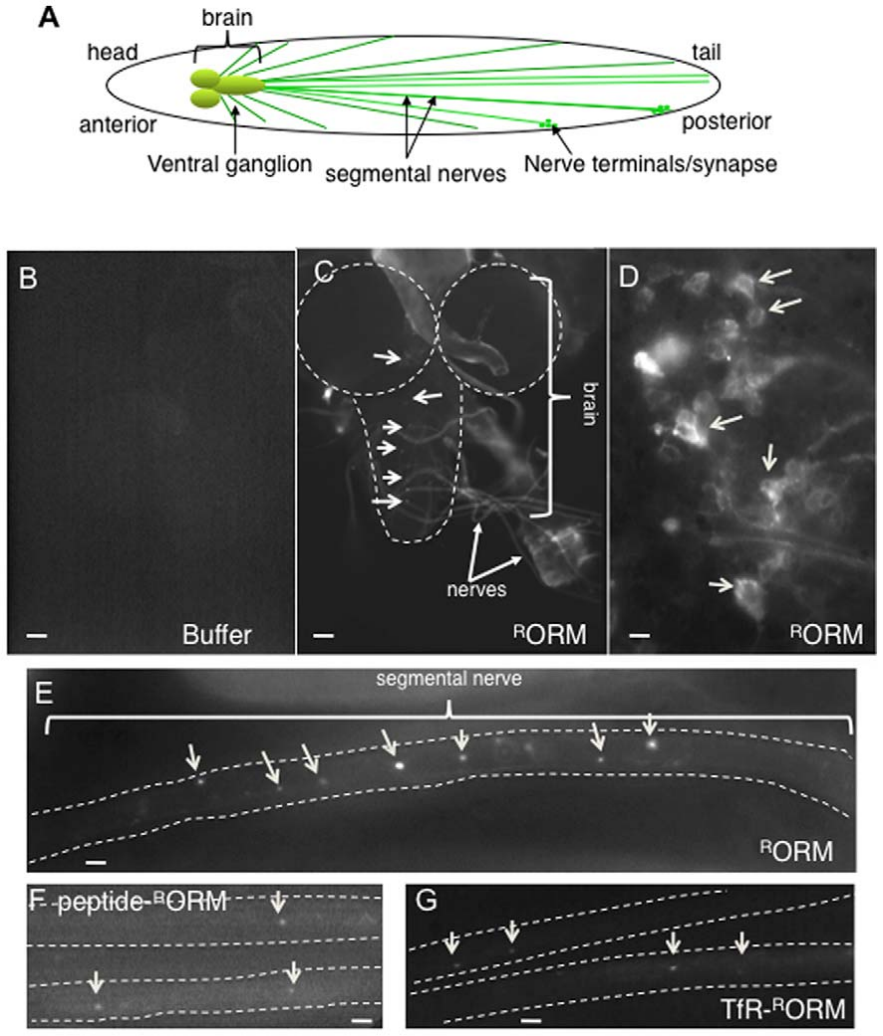
ORMOSIL incorporates into living larval neuronal tissues. (A) A diagram depicting the larval nervous system. The brain is at the anterior. Segmental nerves that originate at the brain extend along the length of the larvae and form synapses or nerve terminals at their ends. Segmental nerves contain bundles of motor and sensory axons, with have their cell bodies located in the brain. (B) Buffer treated larval brain. A representative fluorescence image from a buffer treated larval brain. No fluorescence is observed using the 568 nm filter. Images were taken using the 10× objective. Bar = 10 µm. (C) ORMOSIL is observed within the larval nervous system. A representative low magnification (20×) light microscopy image of a larval brain (dashed outline) and segmental nerves show incorporation of ^R^ORM particles (arrows). ORMOSIL is observed within the brain (outlined, arrows) and the nerves (arrows). Images were obtained using the 568 nm filter to visualize ^R^ORM. Bar = 10 µm. (D) ORMOSIL is observed within the cell bodies of the larval brain. A representative high-magnification fluorescence image using the 100× objective from the ventral ganglion region of an ORMOSIL treated larval brain shows that ^R^ORM is incorporated into the cytoplasm of the neuronal cell bodies (arrows). Note that the cell body nuclei are devoid of ^R^ORM and that the fluorescence signal is in a discrete pattern within the cell body (arrows). Bar = 5 µm. (E) Unconjugated ORMOSIL is observed within the larval segmental nerve. A representative high magnification fluorescence image using the 100× objective from a segmental nerve (outlined) show that ^R^ORM is incorporated into the larval segmental nerve (arrows). ^R^ORM is observed throughout the nerve as bright puncta (arrows). The fluorescence signal we observe can be attributed to accumulated ORMOSIL. Bar = 5 µm (F, G) Conjugated ORMOSIL is observed within the larval segmental nerve. A representative high magnification fluorescence image using the 100× objective from a segmental nerve (outlined) show that conjugated ORMOSIL (Peptide-^R^ORM, F, and TfR-^R^ORM, G) are incorporated into the larval segmental nerves (arrows). Bar = 5 µm.

For a nanoparticle to be successful as a therapeutic tool for neuronal diseases in humans, it must effectively and specifically incorporate into living neuronal tissues and must not cause any adverse effects to living neuronal cells or tissue. To test this, we first evaluated ORMOSIL incorporation into the Drosophila larval neuronal tissues. The Drosophila larval nervous system is a powerful and a well-characterized model system. The central and peripheral nervous systems are remarkably similar in function and in morphology to vertebrates. The larval nervous system is highly organized with the brain at the anterior near the head of the larva (Figure 4A). Segmental nerves extend from the cell bodies in the brain along the length of the larva and these nerves end at the nerve terminals at the distal ends of the larva. Segmental nerves contain bundles of both motor and sensory neurons and are much like the sciatic nerve or the brain stem of mammals.

To determine if ORMOSIL can penetrate and integrate into live larval neuronal tissues, we incubated dissected living larvae with unconjugated and conjugated ORMOSIL (0.2, 0.4 and 1 mg/ml). Larvae were dissected to expose the larval nervous system and incubated in ORMOSIL for 10 minutes. Dissected living larvae were washed in dissection buffer and immediately observed using a confocal microscope under 568 nm fluorescence to visualize ORMOSIL integration into larval neuronal tissues. From our survival experiments we know that ORMOSIL-treated larvae were alive and can survive to over 60 minutes, so our observations were conducted within a 30 min time frame. Unconjugated and conjugated ^R^ORM were found in the larval brain and in the larval segmental nerves (Figure 4). Similarly we also found ^cy5^ORM in the larval brain and larval segmental nerves (Figure S2). The fluorescence we observe was specific to ORMOSIL and was not the result of autofluorescence since buffer or water treated larvae did not show any fluorescence in any of the larval neuronal tissues (Figure 4B and data not shown). In addition, while single nanoparticles are below the resolution of the fluroscence microscope, the fluroscence signal we do observe can be attributed to the accumulations of ORMOSIL nanoparticles at single points. High magnification images taken using a 100× objective indicate that ^R^ORM (or ^cy5^ORM) was present within the cytoplasm of the neuronal cell bodies of the larval brain (arrows, Figure 4D, Figure S2A). Further, ^R^ORM (or ^cy5^ORM) appears to be present in specific cellular compartments and the cell body nuclei appear to be devoid of particles (Figure 4D, Figure S2A). Indeed, in larval cell bodies, some ORMOSIL puncta (Figure S3B, red) colocalized with golgi puncta (Figure S3A, green, merged Figure S3C, arrows). Similar cell body staining was observed for both conjugated forms of ^R^ORM (data not shown). In larval segmental nerves, ^R^ORM (or ^cy5^ORM) was present as puncta along the length of the segmental nerves (Figure 4E, Figure S2B arrows). These puncta may represent small aggregates of ORMOSIL that have accumulated during the 10 min period of nanoparticle incubation. Similarly, Peptide-^R^ORM and TfR- ^R^ORM puncta were also observed in larval segmental nerves (Figure 4F,G arrows). From these observations we can conclude that ORMOSIL can readily incorporate into living larval neuronal tissues perhaps into discrete cellular compartments as suggested previously [26], [41].

**Figure 5:**
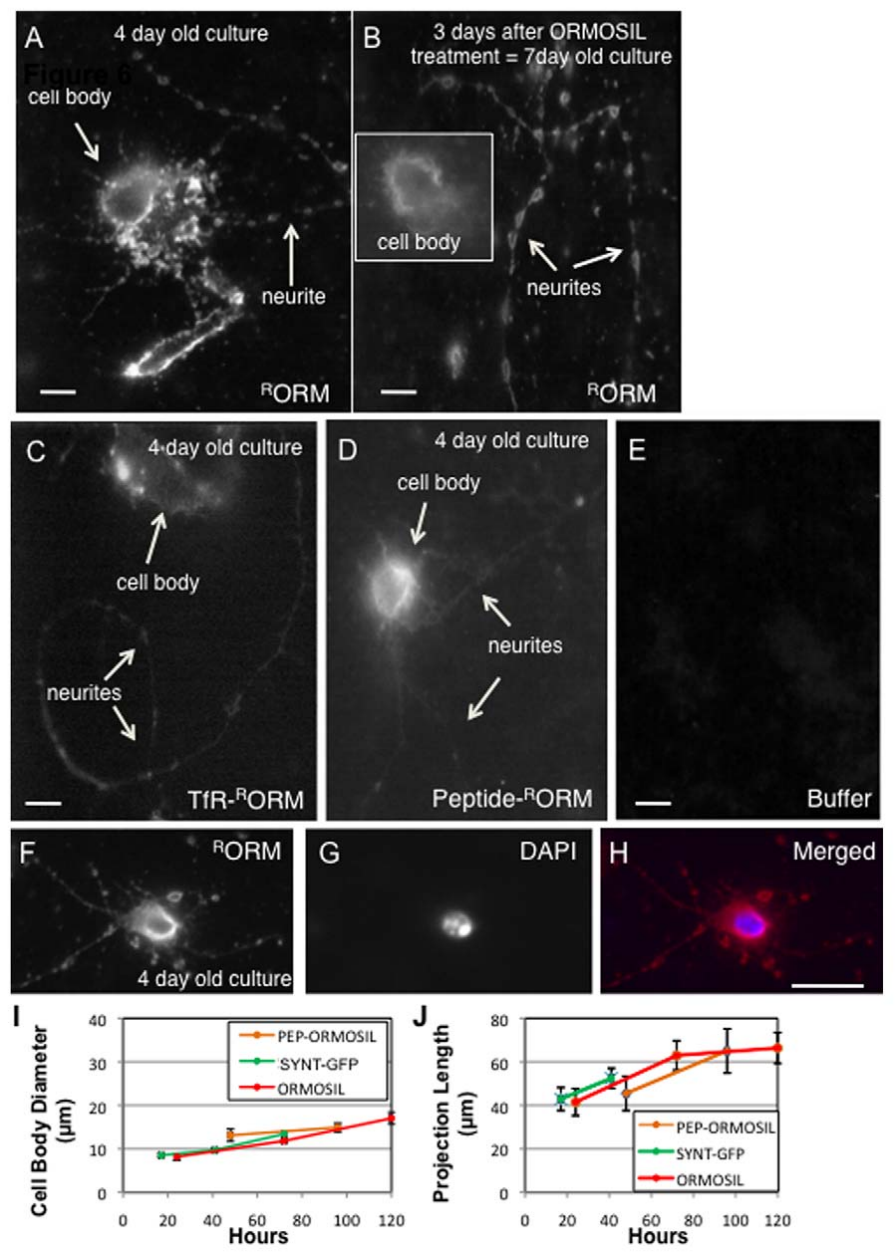
ORMOSIL incorporates into cell bodies and neuritis in primary neuronal cultures. (A) Unconjugated ORMOSIL is observed in 4 day old primary neuronal cultures. 4 day old primary neuronal cultures generated from larval brains were incubated with ^R^ORM. Cultures were imaged using the 568 nm filter. ^R^ORM is observed within the cytoplasm of the cell body in a discrete pattern and within neuritis (arrows). Images were taken using the 100× objective. Bar = 5 µm. (B) Unconjugated ORMOSIL is also observed 3 days after ORMOSIL treatment. To determine how ORMOSIL affected the growth of cultures, ORMOSIL treated cultures were allowed to grow and again imaged after 3 days of ORMOSIL treatment. These cultures were now 7 days old. These treated cultures showed growth with very long neuronal projections compared to day 4. ^R^ORM was still observed within the cell bodies (insert) and the neuritis (arrows,). Note that the fluorescence intensity at day 7 was similar to the intensity observed at day 4. Images were taken using the 100× objective. Bar = 5 µm. (C) Receptor-conjugated ORMOSIL is observed in 4 day old primary neuronal cultures. 4 day old primary neuronal cultures generated from larval brains were incubated with transferrin receptor conjugated ORMOSIL (TfR-^R^ORM). TfR-^R^ORM can be observed within the cell body and within the neuritis. Images were taken using the 100× objective. No significant different in the distribution of ORMOSIL was seen between ^R^ORM and TfR-^R^ORM treated cultures. Bar = 5 µm. (D) Peptide -conjugated ORMOSIL is observed in 4 day old primary neuronal cultures. 4 day old primary neuronal cultures generated from larval brains were incubated with peptide-conjugated ORMOSIL (Peptide-^R^ORM). Peptide-^R^ORM can be observed within the cell body and within the neuritis. Images were taken using the 100× objective. No significant different in the distribution of ORMOSIL was seen between ^R^ORM and peptide-^R^ORM treated cultures. Bar = 5 µm. (E) Untreated control cultures. Control 4 day old cultures treated with buffer do not show fluorescence using the 568 nm filter. Images were taken using the 100× objective. Bar = 5 µm. (F, G, H) ORMOSIL is absent from the cell body nucleus. 4 day old primary neuronal cultures were incubated with ^R^ORM (F, red). Incubated cultures were fixed and stained with DAPI to localize the nuclei (G, blue). A representative merged image (H) indicates that ORMOSIL is absent from the cell body nucleus. Note that ORMOSIL is present in a discrete pattern within the cell body cytoplasm. Images were taken using the 100× objective. Bar = 5 µm. (I) Quantification of cell body growth after ORMOSIL treatment. Graphs depict cell body growth for 120 hours (5 days) after ORMOSIL treatment. For untreated cultures, cultures expressing synaptotagmin-EGFP (SYNT-EGFP) was used. The effect of treatment was evaluated for unconjugated ORMOSIL (ORMOSIL) and for peptide-conjugated ORMOSIL (pep-ORMOSIL). For each time point 8–12 cells from three different experiments were analyzed. The average diameter and standard error was calculated and the average cell body diameter in microns was plotted against time in hours. No statistically significant difference was seen between ORMOSIL treated (unconjugated or peptide-conjugated) and cultures expressing SYNT-EGFP. (J) Quantification of neurite growth after ORMOSIL treatment. Graphs depict neurite growth for 120 hours (5 days) after ORMOSIL treatment. For each time point 8–12 cell from three different experiments were analyzed. The average projection length in micron was plotted against time in hours. The standard error was calculated and plotted. No statistically significant difference was seen in neurite growth between ORMOSIL treated (unconjugated or peptide-conjugated) and cultures expressing SYNT-EGFP.

We also tested ORMOSIL incorporation in living primary neuronal cultures. Primary neuronal cultures were generated from wild type larval brains and allowed to grow for 4 days, when relatively long neuronal projections could be seen. On the fourth day, cultures were incubated with nanoparticles at the three doses (0.2, 0.4 and 1 mg/ml) for 10 minutes. Cultures were washed to remove any unincorporated particles and immediately imaged. Similar to our observations in larval brains, accumulations of ^R^ORM were seen in the cell body and in the neuritis of primary neuronal cultures (Figure 5A, arrows). DAPI staining indicates that the cell body nuclei were also devoid of ^R^ORM particles (Figure 5FGH). Control cultures treated with buffer did not show any fluorescence (Figure 5E). To evaluate if ^R^ORM incorporation caused toxicity to the primary neuronal cultures, and to determine the retention of ORMOSIL in neurons, ^R^ORM treated cultures were allowed to grow for 3 more days (for a total of 7 days) and imaged. ^R^ORM could still be observed 3 days after the initial nanoparticle treatment, in both the cell bodies (Figure 5B, insert) and in the neurites (Figure 5B). The fluroscence intensity was comparable to the initial intensity of ^R^ORM observed 3 days before (compare Figure 5A to 5B). In addition, these neuronal cultures were healthy and the neuritis had grown much longer (about 2 to 3 times longer) during the 3 additional days (Figure 5B). TfR or peptide- ^R^ORM treatment (Figure 5C, D) also showed a similar biodistribition and was comparable to unconjugated-^R^ORM treated primary neuronal cultures. The pattern of unconjugated, TfR or peptide-^R^ORM uptake by these neuronal cells appear to be consistent with previous observations in different cancer cell lines [8], [26], [47], [41], [51]. To further evaluate how ORMOSIL incorporation affected the growth of neurons in culture, we compared and quantified growth by quantifying the changes in the size of the cell body and the changes in the length of neuronal projections for 120 hours (5 days) after ^R^ORM treatment (Figure 5IJ). No statistically significant difference in the growth of either the cell body size or the length of neuronal projections was observed between non-treated cultures (syntaptotagmin-GFP, SYNT-GFP expressing cultures) and ^R^ORM treated cultures (treated with either unconjugated-^R^ORM (cell body, p = 0.728, neuritis, p = 0.698 N = 10–12) or peptide-^R^ORM (cell body, p = 0.737, neuritis, p = 0.861, N = 8–10) during 120 hours after ^R^ORM treatment. In addition no difference in growth was seen in cultures treated with either unconjugated-^R^ORM or peptide-^R^ORM (cell body, p = 0.915, neuritis, p = 0.914, N = 10). Together, these observations suggest that ORMOSIL treatment or incorporation into neurons does not interfere with the normal growth of neurons. Further, ORMOSIL particles are retained within living neurons without any aberrant toxicity.

**Figure 6:**
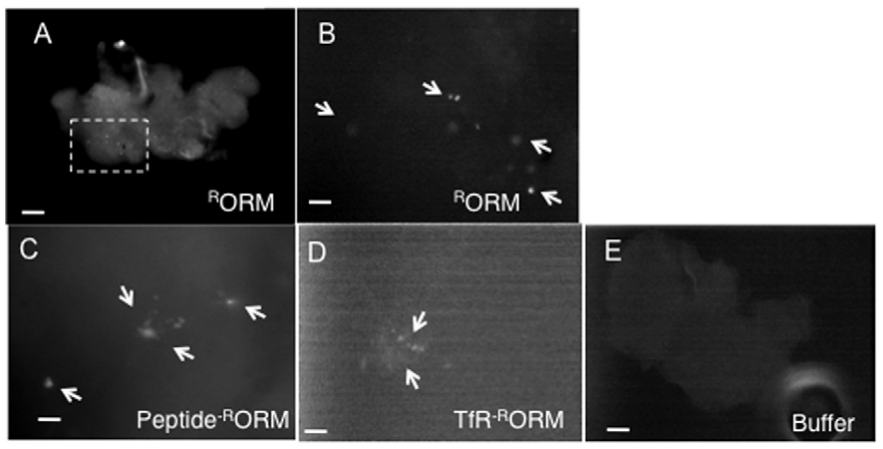
ORMOSIL incorporates into living adult neuronal tissues. (A) Unconjugated ORMOSIL is observed in adult fly brains. A representative fluorescence image of an adult brain from a fly that was fed on ^R^ORM from the time it was a larvae was dissected and observed using the 568 nm filter. ^R^ORM aggregates are observed within the adult brain. Images were taken using the 10× objective. The boxed region is enlarged in B. Bar = 10 µm. (B) Unconjugated ORMOSIL aggregates are observed as puncta within adult fly brains. A high magnification (using the 100× objective) image of the boxed region from A. ^R^ORM aggregates (arrows) are seen as puncta within the adult brain. Interestingly, in all 6 adult brains imaged showed similar puncta of ORMOSIL. Bar = 5 µm. (C) Peptide conjugated ORMOSIL aggregates are observed as puncta within adult fly brains. A representative high magnification image shows peptide-^R^ORM puncta within the adult brain (arrows). Bar = 5 µm. (D) Receptor conjugated ORMOSIL aggregates are observed as puncta within adult fly brains. A representative high magnification image shows TfR-^R^ORM puncta within the adult brain (arrows). Bar = 5 µm. (E) No fluorescence is observed in buffer fed adult fly brains. A representative image from a buffer (1XPBT) fed adult fly brain imaged using the 568 nm filter. Note that there is no fluorescence in these brains. Images were taken using the 10× objective. Bar = 10 µm.

We also evaluated ORMOSIL-fed larvae to determine if these nanoparticles could incorporate into larval neuronal tissues by feeding. We failed to see ORMOSIL within the larval brain (data not shown). However, ORMOSIL was present in many other larval tissues including the larval gut (Figure S1C), malpighian tubes (Figure S1C), trachea (Figure S1B) and skin (cutical, Figure S1B), indicating that these particles can incorporate into larval tissues by feeding. It is possible that ORMOSIL incorporation into neuronal tissues require longer exposure times. In this context, we evaluated adult brains after approx 12–15 days of ORMOSIL exposure. In these adults ORMOSIL was continuously provided by feeding; from the time they hatched into larvae until the time the adults were dissected. Consistent with our hypothesis, we observe unconjugated and conjugated ORMOSIL in dissected adult brains (^R^ORM, Figure 6AB, TfR and peptide-^R^ORM, Figure 6CD), while no fluorescence is observed in brains from control adults (buffer or water fed, compare Figure 6A to 6E). High magnification (100×) images show that ORMOSIL was present in clusters or aggregates within the CNS of the adult brain (Figure 6BCD). These observations were consistent for all 6 adult brains that were imaged (from 3 different feeding experiments). We also noted that the ORMOSIL puncta were consistently in the same region of the brain. These observations suggest that ORMOSIL can be directed into adult neuronal tissues by feeding.

### ORMOSIL nanoparticle incorporation does not activate aberrant neuronal cell death pathways or interfere with normal neuronal processes

**Figure 7:**
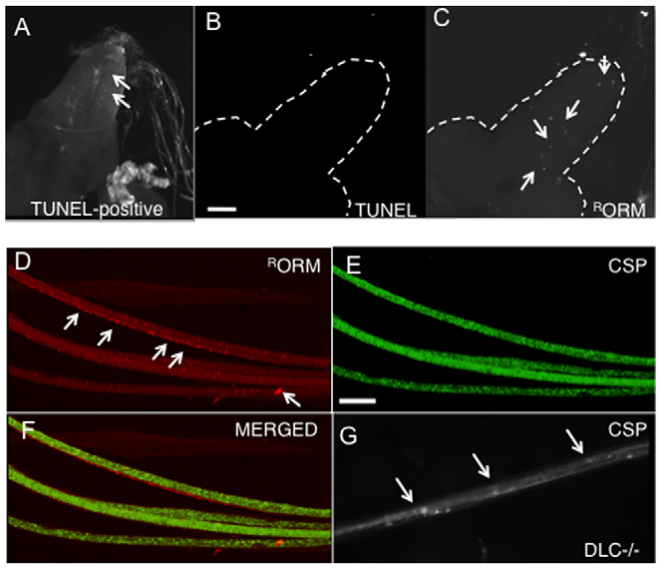
ORMOSIL incorporation does not activate aberrant neuronal cell death pathways or affect the axonal transport pathway. (A, B, C) ORMOSIL nanoparticle incorporation does not induce aberrant neuronal cell death pathways in the cell bodies of the larval brain. A control brain incubated with DNAse and treated with the TUNEL assay shows TUNEL positive cells (A, arrows). A representative fluorescence image from a larval brain (outlined) incubated with ^R^ORM was assayed for neuronal cell death using the TUNEL assay. Using the 488 nm filter, no fluorescence was observed in the larval brain indicating that there were no nuclei showing neuronal cell death (B) in contrast to A. However, ^R^ORM was present within the larval brain (using the 568 nm filter, arrows). Note that ORMOSIL is seen as puncta (C). Images were taken using the 40× objective. Bar = 20 µm. (D, E, F, G) ORMOSIL incorporation does not affect the axonal transport pathway. A representative image of larval segmental nerves from a dissected ^R^ORM treated larvae does not show axonal blockages using the synaptic vesicle antibody, CSP (**E**). Note that ORMOSIL aggregates can be seen in these larval segmental nerves (arrows, **D**). **F** shows the merged image. In contrast, larval segmental nerves from an axonal transport mutant (dynein mutant, DLC) show axonal blockages (arrows, **G**). Images were taken using the 40× objective. Bar = 20 µm.

If these nanoparticles are to be used to develop effective therapeutic tools for human diseases, these particles by themselves should not cause any adverse affects to the target tissue. In this context, we evaluated if ORMOSIL incorporation caused any adverse effects to the normal cellular processes within neurons, such as cell viability and axonal transport. To test this, first, larvae were dissected, treated with ORMOSIL (unconjugated, peptide or TfR-conjugated), fixed and analyzed using the TUNEL assay to evaluate if incorporation of ORMOSIL into neuronal cells activated aberrant neuronal cell death pathways. During cell death, the DNA within the nucleus becomes fragmented and the presence of nicked DNA can be identified by terminal deoxynucleotidyl transferase, an enzyme that catalyzes the addition of dUTPs which can be fluorescently assayed using the TUNEL assay. Thus the presence of fluorescence within nuclei indicates cell death (Figure 7A). We found that ORMOSIL incorporation into larval cell bodies failed to cause aberrant neuronal death. No TUNEL-positive nuclei were observed in ^R^ORM treated larval brains (compare Figure 7B to Figure 7A), although ORMOSIL is observed within the cell bodies (arrows, Figure 7C). Similar observations were seen with conjugated ^R^ORM treated larval brains (data not shown). We further evaluated neuronal cell death in ORMOSIL fed adult brains. Although ORMOSIL puncta were observed in adult brains, there were no TUNEL positive nuclei, indicating that ORMOSIL incorporation by feeding did not trigger abnormal neuronal cell death in adult ORMOSIL-fed brains (unconjugated and conjugated ^R^ORM, data not shown). Thus ORMOSIL incorporation failed to trigger aberrant neuronal cell death pathways.

Secondly, to determine if ORMOSIL incorporation interfered with normal cellular processes within the axon we evaluated the axonal transport pathway using antibodies against the synaptic vesicle marker, cystein string protein (CSP). Within axons, essential components are transported from the cell body, the site where many proteins are synthesized, to the synapse where these components are utilized. This process known as axonal transport is essential for the growth, maintenance and survival of all neurons. Recently, perturbations in this pathway have been implicated in many human neuronal diseases [42], [43]. Previously, we found that segmental nerves from larvae that contain a mutation for a protein involved in axonal transport (i.e., a mutation in the gene encoding the motor protein dynein light chain, DLC) showed bright clusters of CSP accumulations that we call axonal blockages [30], [44] (Figure 7G, arrows), in contrast to wild type larval segmental nerves that show smooth CSP staining [30], [44]. These blockages are packed with many types of identifiable axonal cargo, namely mitochondria, vesicles, large multi-vesicular bodies, and large prelysosomal vacuoles, which interferes with the normal transport of components within the axon [45]. Thus we hypothesized that if ORMOSIL incorporation interfered with the transport of components within the axon then we should observe axonal transport defects similar to these blockages. In contrast to larvae carrying an axonal transport mutation (Figure 7G), ORMOSIL nanoparticle containing larval segmental nerves were smoothly stained (compare Figure 7E to 7G), indicating that the presence of ORMOSIL in larval nerves (Figure 7D arrows) does not cause aberrant axonal transport defects and does not affect the distribution of CSP vesicles. Observations were similar in unconjugated and conjugated forms of ^R^ORM treated larvae (Figure 7 and data not shown). Thus ORMOSIL incorporation does not affect the axonal transport pathway.

**Figure 8:**
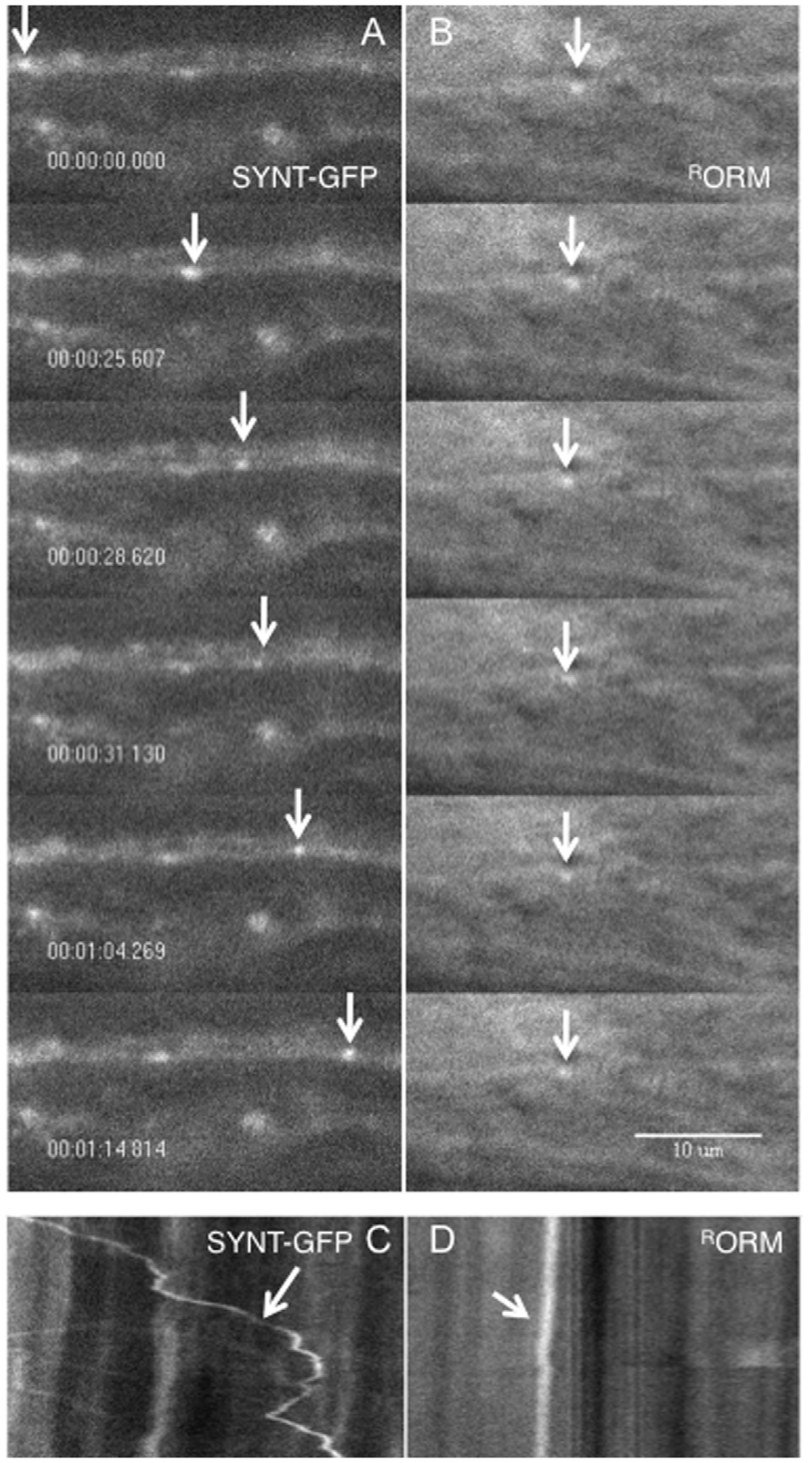
ORMOSIL incorporation does not affect the movement of synaptotagmin vesicles. (A, B) *In vivo* imaging suggests that ORMOSIL incorporation does not affect the movement of GFP-tagged synaptotagmin vesicles within living dissected ORMOSIL treated larvae. Single frames from a representative movie containing both GFP-tagged synaptotagmin vesicles (SYNT-GFP, A) and ^R^ORM (B) simultaneously imaged show the movement of SYNT-GFP vesicles within a larval segmental nerve incubated with ORMOSIL. Note that the SYNT vesicle shows movement in each frame (arrow). (**B**) Note that ORMOSIL is also observed within the same larval segmental nerve (arrow). Note that ORMOSIL does not dramatically change in each frame (arrow). A 1 min movie is depicted. Movies were taken using the 100× objective. Bar = 10 µm. (C,D) Kymographs show that ORMOSIL treatment does not affect the movement of GFP-tagged synaptotagmin vesicles. Kymographs from the above movies show that the movement dynamics of SYNT-GFP vesicles are not affected in ^R^ORM treated larvae. The movement of a SYNT-GFP vesicle is seen moving from the left to the right (arrow, C), while a slight shift is seen for ORMOSIL (arrow, D).

We further investigated if ORMOSIL incorporation affected the *in vivo* transport dynamics of vesicles within larval axons in whole-mount larvae. Larvae expressing a GFP tagged vesicle protein (synaptotagmin-EGFP, SYNT-EGFP) were dissected and treated with ORMOSIL particles (unconjugated or conjugated) at doses 0.2, 0.4 or 1 mg/ml. Simultaneous observations using dual view imaging, a beam splitter and split view software showed the movement of the GFP-tagged synaptotagmin vesicles (Figure 8AC) within larval axons, together with ORMOSIL (Figure 8BD, data not shown). The movement dynamics of GFP-tagged synaptotagmin vesicles was not altered by the incorporation of ORMOSIL (both unconjugated and conjugated) at these doses, and was comparable to the movement dynamics of GFP-tagged synaptotagmin vesicles in untreated larvae [40]. Similar observations were also seen in primary neuronal cultures generated from larval brains expressing SYNT-GFP treated with ORMOSIL (unconjugated and conjugated, data not shown). Thus our observations indicate that ORMOSIL incorporation at the concentrations we used does not interfere with the movement of synaptic vesicles *in vivo*, an essential pathway for neuronal integrity and survival.

## Discussion

For any nanoparticle to be useful as a therapeutic delivery system, it must first pass three basic criteria; it should not be toxic to the organism, it should effectively incorporate into the specific tissue type that the treatment targets and it must not interfere with the normal functions of that tissue type. Our study demonstrates that a novel silica based particle named ORMOSIL successfully passes all three of these tests and could have enormous potential for the development of a therapeutic system for living neurons within whole organisms.

Until now, only viral carriers have been successfully used in treatment strategies in animal models of disease and injury [47], [48], but safety concerns relating to the toxicity and immunogenicity of viral vectors remain a challenge to clinical translation [49]. Non-viral delivery vehicles such as ORMOSIL have the potential to avoid the safety issues inherent in viral delivery, but lack of knowledge about their behavior in neuronal tissues and how these particles affect normal cellular processes within a whole organism has so far limited their use. Our study tested the behavior of both unconjugated and receptor or peptide conjugated ORMOSIL in whole living organisms, throughout development, and in living neuronal cells. We found that these novel particles are biocompatible, are not cytotoxic and incorporate into neuronal tissues. Thus these particles have great potential to be effectively translated for used in neuronal therapeutic applications.

Unlike other nanoparticles, these nanoparticles have several physiochemical properties that facilitate their use in many biomedical applications [8]. They are easily synthesized in a cost-efficient manner, have excellent storage stability and are resistant to contamination. They are optically transparent and are excellent optical probes following encapsulation or conjugation of flurophores because nano-incorporation protects flurophores from photobleaching and prevents interaction with the biological environment [10]. They have also been extensively used as probes for imaging in *in vitro* experiments [13]. Further, the porus surface of ORMOSIL allows co-incorporation of other diagnostic probes for recognition of molecules for target-specific delivery without changing its size [11], [26].

Previous studies using ORMOSIL nanoparticles in a pancreatic cancer cell culture system showed no indication of cytotoxicity and good uptake of these particles was observed using confocal microscopy [8], [19]. Further, fluorescence anisotropy experiments demonstrated that the flurophores were still coupled to the particles. Robust uptake was also detected with ORMOSIL particles conjugated with various biotargeting molecules with no cytotoxicity. Although studies using systemic injection in mice have been performed, these studies failed to assess the long-term toxicity of ORMOSIL to the animal or to the animal's tissues [26], [18]. Thus we chose Drosophila as a model organism to test toxicological effects and to obtain dosage information because the fly has a rapid life cycle and the maintenance costs are relatively low. Our observations in a whole organism indicate that ORMOSIL is not toxic to developing Drosophila. Oral administration of ORMOSIL by feeding to Drosophila, (through its development) failed to show any developmental defects or induced lethality. Phenotypically and behaviorally larvae and adults continuously fed on ORMOSIL were undistinguishable from those fed on water or buffer. Further more, our feeding experiments used doses of ORMOSIL that were much higher than what has previously been used in intravenous experiments in mice [26], [18]. We also failed to see any behavioral defects or any abnormalities to the adult lifespan in flies that were continuiously fed ORMOSIL. Importantly, different nanoparticle formulations with different fluorescent tags and different biotargeting tags showed very similar behaviors, uptake patterns and distributions inside living animals and living neuronal cells. Thus these novel particles appear to be good candidates for the development of therapeutics in a whole organism. Further, our study is the first to test the biocompatibility of a nanoparticle through the development of an organism and through long-term administration. A recent invertebrate study in C. elegans examined the effects of a different kind of nanoparticle in adults, but failed to determine its effects through the developmental cycle of C. elegans [46].

Recently the biodistribution of ORMOSILwas tested by systemic injection in mice [26]. In these studies 2.0 mg per kg of body weight of ORMOSILwas injected into the tail vein and the incooperation of particles were followed in different tissues. Almost 75% of the injected dose was observed in the liver, spleen and skin, whereas the lung, kidney and the heart accounted for less than 5% of the injected dose. However, no particles were seen in neuronal tissues. Although our feeding experiments also show ORMOSIL in the gut and skin, in contrast, we found ORMOSIL incorporation in the adult brain, indicating that continuous ingestion of ORMOSIL for an extended period of time can lead to the incorporation of these particles into the adult fly brain. Although further study is needed to elucidate the exact mechanism of incorporation, our observations propose that single ORMOSIL particles may penetrate the Drosophila blood-brain barrier [50] and form ORMOSIL aggregates. Since penetration of the blood-brain barrier is an important requirement for an effective therapeutic particle further study is needed to evaluate the exact mechanism.

Our observations also indicate that ORMOSIL is an attractive new particle for the development of therapies specifically targeted to neuronal tissues. Previous studies have proposed that silica based nanoparticles can be readily taken up by the cell as individual entities mostly by phagocytosis or receptor mediated uptake followed by accumulation in specific cellular compartments [26], [41]. We also found that ORMOSIL particles can readily incorporate into the cytoplasm of the neuronal cell bodies and to larval axonal projections or neuritis. The puncta of fluorescence we observe (Figure 4E, 6B) can be attributed to the fluorescence from ORMOSIL particles, which entered the cells as individual entities and accumulated. Incorporation also appears to be to a specific cellular compartment (Figure S3) and ORMOSIL was absent from the neuronal cell body nuclei (Figure 5). Importantly, since both unconjugated and peptide or receptor conjugated ORMOSIL was seen in cell bodies and neuritis, conjugation of ORMOSIL does not appear to change the localization of ORMOSIL within the neuronal cell, although the mechanism of uptake may be different for unconjugated (by phagacytosis) and receptor/peptide conjugated (receptor mediated) ORMOSIL [26], [41]. Further study will be needed to elucidate this proposal.

We also observe that the presence of ORMOSIL within neurons did not activate aberrant neuronal cell death pathways or interfere with normal neuronal processes such as axonal transport. Incorporation of ORMOSIL did not induce neuronal cell death. Further, incorporation of ORMOSIL into living larval axons or neuritis did not affect the movement dynamics of synaptic vesicles. Thus while ORMOSIL can affectively incorporate into neurons these particles do not appear to interfere with normal neuronal processes, two important requirements for an effective therapeutic particle.

In conclusion, our study demonstrates that ORMOSIL nanoparticles have great promise for the development of therapeutic applications for human neuronal disease for long-term use within whole organisms. Its non-toxic characteristic and its ability to readily incorporate into living neuronal tissues together with its porous surface that allows incorporation of specific molecules for targeting, make ORMOSIL nanoparticles the next generation of particles that can be effectively used to develop targeted therapeutic treatments to specific areas of the brain or to specific populations of neurons. Development of such treatment strategies have great potential in minimize global deleterious affects while maximizing beneficial affects, which is a problem in many of the current treatment strategies that are used for many human neuronal diseases.

## Materials and Methods

### Synthesis of ORMOSIL nanoparticles

ORMOSIL nanoparticles with covalently incorporated fluorophores, rhodamine or cy5, were synthesized in the non-polar core of an oil-in-water microemulsion, as per our earlier report but with a slight modification [8]. Briefly, 0.2 g of surfactant AOT and 300 µl of n-butanol and 100 µl of DMSO were dissolved in 10 ml HPLC grade water by magnetic stirring. To this microemulsion system, 100 µl of 1 mg/ml of rhodmaine-silane precursor (rhodamine NHS ester, conjugated to aminopropyltriethoxy silane) in DMSO was added and stirred, followed by an addition of 100 µl of neat VTES. The reaction mixture was stirred for another hour and polymerization reaction was started by the addition of 10 µl of amino-propyltriethoxysilane. The final reaction mixture was allowed to stir overnight. The mixture was then dialyzed against distilled water for 48 h at room temperature, using a cellulose membrane with a cut-off size of 12–14 kD. Following dialysis, the nanoparticles were sterile filtered and stored at 4°C for future use. Using the oil-in-water microemulsion method ^R^ORM was conjugated with the bioactive molecules transferrin (Sigma-Aldrich, [8]) and the 15 aa peptide (G-Y-E-N-P-T-Y-K-F-F-E-Q-M-Q-N, Celtek peptide, [29]). As described previously [8], 1 mL of stock solution of -COOH-terminated ^R^ORM nanoparticles was mixed with 25 µl of 0.1 M EDC solution and gently stirred for 30 min. Next, 5 µl of 5 mg/mL of transferrin or 5 µl of 5 mg/ml of peptide was added into this mixture, and the mixture was incubated at room temperature for 2 h to allow the proteins to covalently bond to the COOH group of the ORMOSIL nanoparticles. The conjugation was confirmed by agarose gel electrophoresis by measuring the ζ-potential values of the resulting nanoparticles [8].

### Drosophila feeding experiments

Wild type Drosophila cages were used to collect embryos for ORMOSIL (unconjugated or receptor or peptide conjugated) feeding experiments. Eggs were collected on egg laying apple plates for 12 hours at room temperature. Using a brush the eggs were collected into a fine sieve, washed and placed in a microcentrifuge tube in a suspension of water. 200 µl of egg/water solution was placed in each vial that contained dry food (Instant Medium, Carolina Biological, which is a commonly used food for Drosophila), mixed with the three different doses of ORMOSIL (0.2, 0.4 and 1 mg/ml), 1X PBT (Phosphate Buffered Saline (PBS) containing triton X-100) buffer or water. 1 mg/ml ORMOSIL were diluted to 0.2 and 0.4 mg/ml using 1X PBT buffer. Similar procedures were previously used in a chemical screen that tested 500 small molecular compounds to identify chemical modifiers of axonal blockages (Gunawardena unpublished). In this screen we found that feedings using concentrations of 1 mg/ml of modifier compound was very toxic to developing Drosophila (Gunawardena unpublished). Thus we determined that concentrations of 1 mg/ml and below were ideal for nanoparticle feeding experiments in Drosophila.

Vials containing food laced with the different forms of ORMOSIL, buffer or water were kept in the dark in a humid chamber and the larval, pupae and adult development was observed at room temperature (25°C). At each developmental stage of the Drosophila life cycle, larvae, pupae and adults were counted. The larvae were counted as the larvae crawled up the vial. The pupae were counted as the larvae pupated. As adults eclosed from pupae they were counted and placed in a new vial that contained the corresponding concentration of ORMOSIL, buffer or water. The survival of adults was assayed for over 100 days.

To assay for ORMOSIL incorporation, ORMOSIL fed whole 3^rd^ instar larvae were mounted on a slide and imaged under a Nikon TE 2000 fluorescent microscope. For visualization of ^R^ORM/^cy5^ORM the 568/680 nm filter was used to observe rhodamine distribution within the larva (Figure S1BC). As controls buffer fed and water fed larvae were also imaged. These larvae did now show any fluorescence in the 568 nm/680 nm filter (Figure S1DE and data not shown). For each condition over 10 larvae were imaged. Visualization was also done using the 488 nm filter. Images were also taken in phase contrast to visualize the whole larvae (Figure S1A).

### Quantification of survival of larvae, pupae and adults

To quantify the survival of ORMOSIL and control fed larvae we counted and labeled larvae using a colored pen. Each day new larvae were counted and recorded were recorded for each experimental condition. As the larvae pupated each pupae was counted and labeled using a different colored pen to determine the survival of pupae. Each day new pupae were counted and the total numbers of pupae were recorded for each condition. Further, as adults emerged they were collected, counted and placed in new vials containing food laced with the appropriate feeding condition. This day was recorded as Day 0. More than 100 flies from 3 experiments were pooled for quantification and graphing. For each feeding condition the total numbers of pupae and adults were normalized and graphed as percent survival (Figure 2B). Once adults started eclosing the numbers of adult flies eclosing on 3 constisutive days were counted and normalized to determine if there was a delay in development between the different feeding conditions (Figure 2C). To generate the survival curve the number of dead adults were counted on day 18, 25 and 33. These numbers were subtracted from the total number of flies on day 0 and normalized and plotted as percent survival (Figure 2D). Note that survival of adult flies was counted for a total of 33 days, although vials were evaluated for over 100 days. Student T-test was used to determine statistically significant differences between the feeding conditions. Graphs were plotted using an EXCEL spreadsheet.

### Larval dissections and adult brain dissections

Larvae were dissected as described previously in 1× dissection buffer, under physiological conditions [30], [36]. Usually during dissections, the buffer is replaced every 20–30 minutes to keep the physiological conditions constant and under these conditions dissected larvae survive for over 180 minutes. However, since we were evaluating the effect of ORMOSIL on dissected larval survival, the buffer was not changed. Under these modified conditions the dissected larvae survived for more than 60 minutes. ORMOSIL or dissection buffer was added to living dissected larvae and incubated for 10 minutes at room temperature in the dark. After treatment the living dissected larvae were washed with dissection buffer and mounted for immediate observation using a Nikon TE 2000 fluorescent microscope using the 568 nm filter or a Leica TCS SP2 AOBS spectral confocal microscope (Leica Microsystems Semiconductor GmbH, Wetzler, Germany) using the krypton/argon laser. Images were obtained using a 100× or 63× objective.

The survival of dissected living larvae incubated with ORMOSIL or dissection buffer was quantified using “survival points”. “Survival points” were assigned according to the extent of twitching observed when the larva was stimulated with a forceps [37], [38], [39]. The higher the survival value the more the larva twitched, indicating that the larva was alive. The lower the survival value the less the larva twitched and was more likely to be dying. The twitching phenotype or the twitch tension is a commonly used phenotype in Drosophila larvae and evaluates nerve-stimulated contractions of larval body-wall muscles [37], [38], [39]. For each condition the survival of 10 treated larvae was followed for 60 minutes. The extent of twitching for each larva was recorded every 15 minutes for a total of 60 minutes. Graphs were plotted using an EXCEL worksheet, with the Y axis depicting the larval survival and the x axis depicting the time in minutes (Figure 3).

Adult flies fed on ORMOSIL or control conditions were aged for 10 days and their brains were dissected using 1× dissection buffer. 6 dissected brains for each feeding condition were immediately mounted and imaged using the 568 nm filter to image for ORMOSIL incorporation using a 20× and 40× objective.

### Immunohistochemistry, TUNEL assay and Confocal Imaging

Dissected larvae were treated with ORMOSIL, buffer or water as described above and fixed in 4% formaldehyde. Immunohistochemistry was performed as previously described in [30], [44], [36]. Briefly, fixed larvae were washed in 1XPBT and incubated overnight with antibodies against CSP (cysteine string protein, a synaptic vesicle protein, Developmental Studies Hybridoma Bank), to assay for axonal blockages at a dilution of 1∶10. The primary antibody were incubated overnight at 4°C. The secondary antibody Alexa 488 anti-mouse (Invitrogen) was used at a concentration of 1∶200 since ORMOSILwas imaged using the 568 nm filter. The secondary antibody was incubated for one hour at room temperature in the dark. After washing with 1XPBT, larvae were mounted on slides using vectorshield mounting medium for confocal microscopy using a Leica TCS SP2 AOBS spectral confocal microscope (Leica Microsystems Semiconductor GmbH, Wetzler, Germany). Images were taken using a 63× oil immersion objective using the krypton/argon laser to image the synaptic protein CSP in 488 nm and ORMOSIL in 568 nm.

To evaluate cell death, the TUNEL assay (Roche) was performed as previously described in [30], [44]. Larval brains were dissected in 1× dissection buffer and incubated in ORMOSIL similar to what was done in larval dissections. Larval brains were fixed using 4% formaldehyde and the TUNEL assay was performed as per manufactures instructions. Negative (no substrate) and positive (DNAse digestion of larval brains, Figure 7A) were done as controls to test the sensitivity of the assay. Larval brains were mounted using vectorshield mounting medium and imaged using the confocal microscope using the krypton/argon laser to assay for cell death in 488 nm and ORMOSIL incorporation in 568 nm. Images were taken using the 63× oil immersion objective and the Leica TCS SP2 AOBS spectral confocal microscope.

### In vivo imaging of GFP-tagged vesicles and ORMOSIL in dissected living larval segmental nerves

GFP-expressing larvae were generated by crossing males that carried the transgene synaptotagmine-EGFP (UAS-SYNT-EGFP) to female virgins that carried the transgene APPL-GAL4 as described in [44], [40]. In the progeny the activator GAL4 binds to the GAL4 binding sites of UAS-SYNT-EGFP thus expressing the synaptotagmin-EGFP vesicle protein. GAL4 expression is controlled by a neuronal specific promoter (APPL) thus SYNT-EGFP expression only occurs in neuronal tissues. Synaptotagmin is a vesicle protein. GFP expressing larvae are dissected and incubated with ORMOSIL as described above. After ORMOSIL incubation (10 minutes), ORMOSIL was washed using dissection buffer. These dissected larvae are immediately imaged using the 100× oil immersion objective. Using the beam splitter and split view software simultaneous movies are obtained for GFP (488 nm filter) and ^R^ORM (568 nm filter), using the Nikon TE 2000 fluorescent microscope. Simultaneous movies of GFP vesicles and ORMOSIL particles containing 100 frames were taken at 150 msec as previously done [44], [40]. Kymographs of each movie are generated using MetaMorph software.

### ORMOSIL treatment in primary neuronal cultures and quantification of neuronal growth

Primary neuronal cultures were generated from wild type 3^rd^ instar larval brains as previously described [51]. Briefly, larval brains are dissected, washed with ethanol and incubated in a Liberase enzyme solution (17 ul liberase solution and 1 mL of Ranaldini's Saline) for 1 hour at 29 degrees. The enzyme is replace by medium and the medium is replace three times. After the last replacement with medium, the neurons are resuspended with a pipette to break the cells. The suspension of cells is pipetted onto culture dishes containing glass coverslip bottoms and incubated at 29 degrees. Cultures are flooded with flooding medium (S10-I medium and 20E hormone). The cultures are allowed to grow for 4 days at 29 degrees. On the 4^th^ day, the flooding medium is replaced with the flooding medium containing different forms of ORMOSIL (0.2 mg/ml, 0.4 mg/ml and 1 mg/ml) or buffer for 10 minutes at 29 degrees. After 10 minutes the cultures are washed with flooding medium and immediately imaged using the Nikon TE 2000 fluorescent microscope, the 100× oil immersion objective and the 568 nm filter. After imaging the cultures are allowed to grow for another 3 days at 29 degrees. These cultures (7 days old) are again imaged to evaluate the growth of these cultures 3 days after ORMOSIL incorporation. The growth in ORMOSIL treated cultures is evaluated by comparing the lengths of neuritis in ORMOSIL treated cultures to non-treated cultures. Both ORMOSIL treated and non-treated cultures showed 2–3 times longer neuritis at 7 days than 4 days. 4 day old cultures were also treated with Transferrin and peptide-conjugated ORMOSIL as described above.

To quantifying neuronal cell growth in cultures the diameter of the cell body and neurite length were measured. For neurite length the longest projection was used. Quantification was done in untreated cultures expressing SYNT-EGFP and in cultures treated with unconjugated and peptide conjugated ORMOSIL. All measurements were taken in MetaMorph using the multi-line tracing tool. To measure the cell body diameter, a linear line was traced through the cell body end to end. In the case of asymmetrical cell bodies, two lines were used perpendicular to each other and averaged to get the diameter. To measure the neurite length, one line was traced along the length of the longest neurite. Any cells lacking visible neurites were omitted from the analysis. Measurements were obtained from 8 to 12 individual cells and averaged. Data was plotted on an Excel worksheet and the standard error and p values were calculated.

## Supporting Information

Figure S1 **ORMOSIL conjugated with Cy5 also penetrates into larval cell bodies and larval segmental nerves.** (**A**) ^Cy5^ORM treated larvae show ORMOSIL in larval cell bodies (arrows), similar to figure 4D. Note that the cell body nuclei are devoid of ORMOSIL. Bar = 5 µm. (**B**) A representative high magnification fluorescence image from a segmental nerve (outlined) shows that ^cy5^ORM is also incorporated into the larval segmental nerve (arrows). ^cy5^ORM is observed throughout the nerve as bright puncta (arrows). The fluorescence signal we observe can be attributed to accumulated ORMOSIL. Bar = 5 µm.
(TIF)

Figure S2 **ORMOSIL nanoparticles incorporate into living larval tissues.** (A) A composite phase contrast image of a living larva. The head is at the anterior end while the tail is at the posterior end. These images of whole larvae were constructed by putting together several images taken using the 10× objective. (B) A composite fluorescence image of an ORMOSIL fed living larva imaged for ^R^ORM using the 568 nm filter. ORMOSIL nanoparticles can be observed within the skin (cuticle) and trachea (anterior posterior lines). Bar = 100 µm. (C) A composite fluorescence image of an ORMOSIL fed living larva imaged for ^R^ORM. Note that ORMOSIL nanoparticles are observed as puncta and are present in the guts and malpighian tubes. (D) A composite fluorescence image of a control, buffer (1XPBT) fed living larva imaged using the 568 nm filter. Note that no distinct fluorescence is observed. (E) A composite fluorescence image of a control, water fed living larva imaged using the 568 nm filter. No obvious fluorescence is observed. The guts show some auto fluorescence, but note that the staining pattern is different from what is seen in C and B.
(TIF)

Figure S3 **ORMOSIL is present within specific cellular compartments.** (A) A representative image showing cell bodies from ORMOSIL treated larval brains stained with the golgi marker gp120 (green). (B) ORMOSIL can be observed in red. Note that some ORMOSIL puncta and golgi puncta colocalize (arrows in merged image, C) indicating that ORMOSIL is present within discret compartments within the cell body and is not at the cell surface. Bar = 5 µm.
(TIF)
