# Supplementary figures and images for: Organically Modified Silica Nanoparticles Are Biocompatible and Can Be Targeted to Neurons In Vivo
Source: PLoS One. 2012 Jan 3;7(1):e29424. doi: 10.1371/journal.pone.0029424 (PMC3250438; doi:10.1371/journal.pone.0029424)

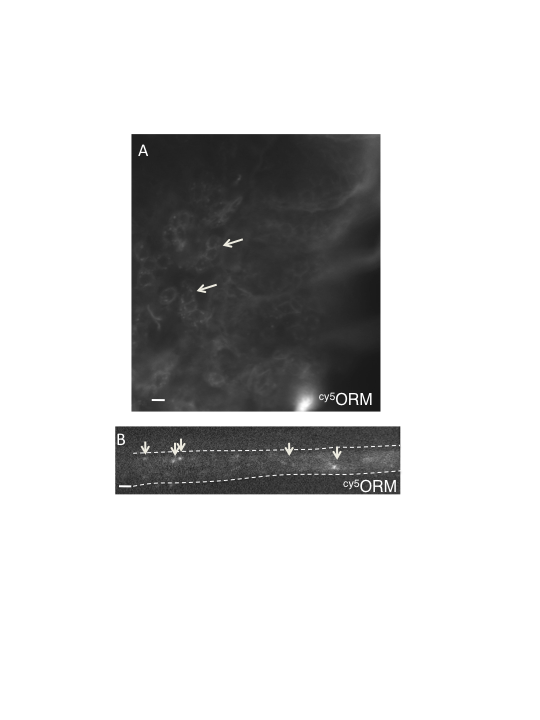

Supplement: Figure S1 — ORMOSIL conjugated with Cy5 also penetrates into larval cell bodies and larval segmental nerves. (A) Cy5ORM treated larvae show ORMOSIL in larval cell bodies (arrows), similar to figure 4D. Note that the cell body nuclei are devoid of ORMOSIL. Bar = 5 µm. (B) A representative high magnification fluorescence image from a segmental nerve (outlined) shows that cy5ORM is also incorporated into the larval segmental nerve (arrows). cy5ORM is observed throughout the nerve as bright puncta (arrows). The fluorescence signal we observe can be attributed to accumulated ORMOSIL. Bar = 5 µm. (TIF) [file pone.0029424.s001.tif]

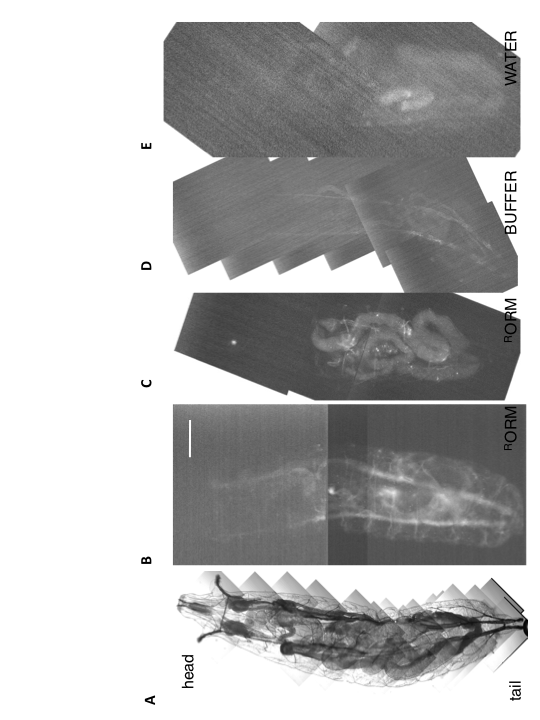

Supplement: Figure S2 — ORMOSIL nanoparticles incorporate into living larval tissues. (A) A composite phase contrast image of a living larva. The head is at the anterior end while the tail is at the posterior end. These images of whole larvae were constructed by putting together several images taken using the 10× objective. (B) A composite fluorescence image of an ORMOSIL fed living larva imaged for RORM using the 568 nm filter. ORMOSIL nanoparticles can be observed within the skin (cuticle) and trachea (anterior posterior lines). Bar = 100 µm. (C) A composite fluorescence image of an ORMOSIL fed living larva imaged for RORM. Note that ORMOSIL nanoparticles are observed as puncta and are present in the guts and malpighian tubes. (D) A composite fluorescence image of a control, buffer (1XPBT) fed living larva imaged using the 568 nm filter. Note that no distinct fluorescence is observed. (E) A composite fluorescence image of a control, water fed living larva imaged using the 568 nm filter. No obvious fluorescence is observed. The guts show some auto fluorescence, but note that the staining pattern is different from what is seen in C and B. (TIF) [file pone.0029424.s002.tif]

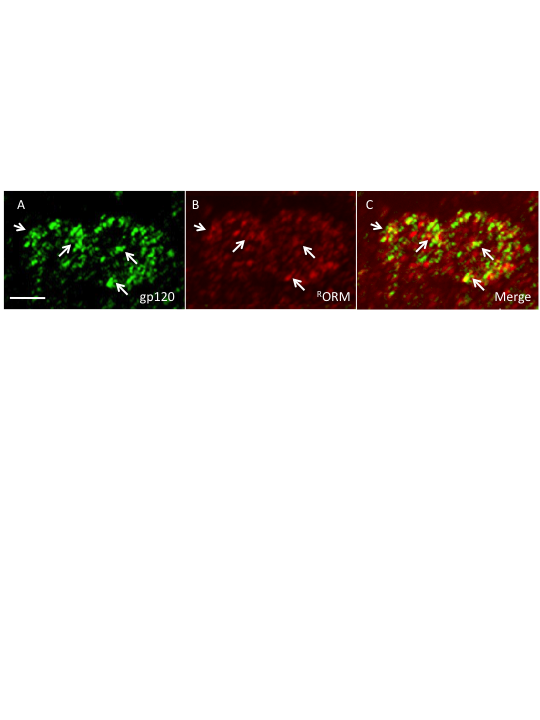

Supplement: Figure S3 — ORMOSIL is present within specific cellular compartments. (A) A representative image showing cell bodies from ORMOSIL treated larval brains stained with the golgi marker gp120 (green). (B) ORMOSIL can be observed in red. Note that some ORMOSIL puncta and golgi puncta colocalize (arrows in merged image, C) indicating that ORMOSIL is present within discret compartments within the cell body and is not at the cell surface. Bar = 5 µm. (TIF) [file pone.0029424.s003.tif]
